# Severe DRESS syndrome with hemophagocytic lymphohistiocytosis and cryptococcal meningitis: a case report and diagnostic challenge

**DOI:** 10.3389/fimmu.2026.1703040

**Published:** 2026-01-30

**Authors:** Shi Yan, Yu-ting Gan, Jia-jia Li, Yu Sheng, Bing-jun Shi

**Affiliations:** 1 Department of Dermatology, Chongqing Hospital of Traditional Chinese Medicine, Chongqing, China; 2 Chongqing Key Laboratory of Integrative Dermatology Research, Chongqing, China

**Keywords:** diagnostic, DRESS (drug reaction with eosinophilia and systemic symptoms), hemophagocytic lymphohistiocytosis (HLH), meningitis, treatment

## Abstract

We report a rare and severe case of drug reaction with eosinophilia and systemic symptoms (DRESS) in a 72-year-old woman, complicated by hemophagocytic lymphohistiocytosis (HLH) and cryptococcal meningitis. The patient initially presented with fever, rash, eosinophilia, and liver dysfunction following antibiotic exposure. Despite corticosteroid therapy, she developed progressive cytopenias and neurological symptoms. Further evaluation confirmed HLH and cryptococcal infection. Treatment included intravenous immunoglobulin, antifungal agents, and multidisciplinary supportive care, resulting in gradual recovery. This case highlights the diagnostic complexity of overlapping immunologic and infectious conditions and underscores the importance of early recognition and coordinated management in patients with severe drug hypersensitivity reactions.

## Introduction

Drug reaction with eosinophilia and systemic symptoms (DRESS) is a rare, severe drug-induced hypersensitivity syndrome characterized by skin eruptions, eosinophilia, and internal organ involvement (1). Visceral involvement is a hallmark of severe DRESS, extending beyond the commonly affected liver to include the kidneys, lungs, heart, and gastrointestinal tract (2). Renal manifestations, such as acute interstitial nephritis and acute kidney injury, occur in approximately 10%–30% of patients and are associated with worse clinical outcomes (3). Although less common, pulmonary involvement—including interstitial pneumonitis and pleural effusion—along with cardiac complications, such as myocarditis and pericarditis, are critical determinants of mortality and require prompt recognition and intervention. Additionally, gastrointestinal involvement, manifesting as hepatitis, pancreatitis, or colitis, further exacerbates the systemic inflammatory burden and complicates the clinical course (3). Although uncommon, DRESS can lead to hemophagocytic lymphohistiocytosis (HLH), a potentially fatal hyperinflammatory condition (4). Secondary HLH, also known as macrophage activation syndrome, is a life-threatening condition characterized by uncontrolled immune activation. It is most commonly triggered by infections—particularly viral pathogens—malignancies, and autoimmune diseases. Moreover, severe drug reactions, such as DRESS, are now well recognized as significant precipitants, as highlighted in recent diagnostic guidelines (5). Opportunistic infections may further complicate the course, particularly under immunosuppressive therapy. Here, we report a case of DRESS complicated by secondary HLH and cryptococcal meningitis in an elderly diabetic woman—to our knowledge, the first reported case of this triad—highlighting the diagnostic and therapeutic challenges in such overlapping immune dysregulation.

## Case description

**Figure 1:**
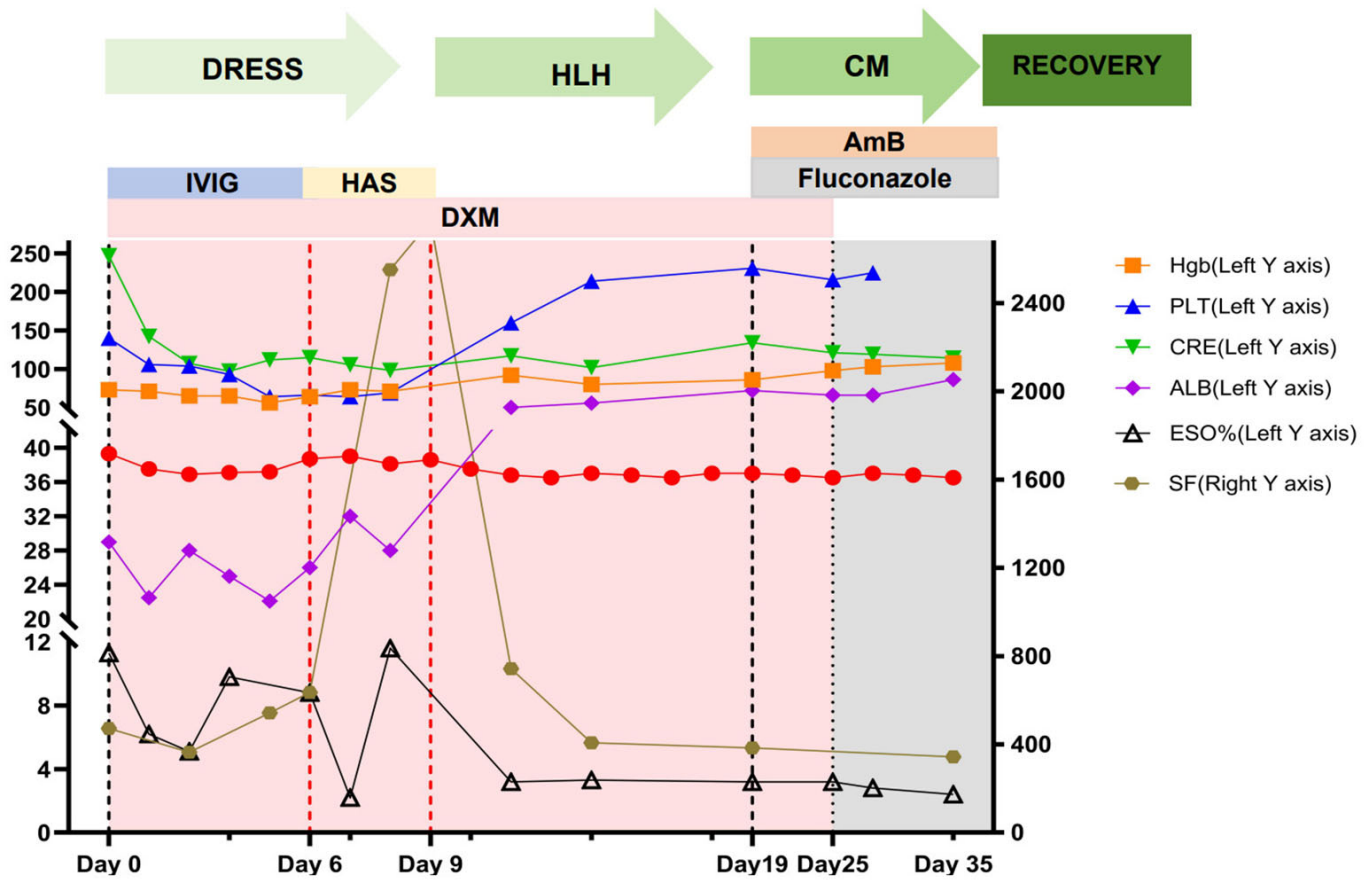
Changes in patient’s treatment and tests. MPSS, methylprednisolone sodium succinate; HAS, human albumin solution; IVIG, intravenous immunoglobulin; DXM, dexamethasone; AmB, Amphotericin B; T, temperature; Hgb, hemoglobin; PLT, platelet count; CRE, creatinine; ALB, albumin; ESO%, eosinophil percentage; SF, serum ferritin; IgE, Immunoglobulin E.

**Figure 2:**
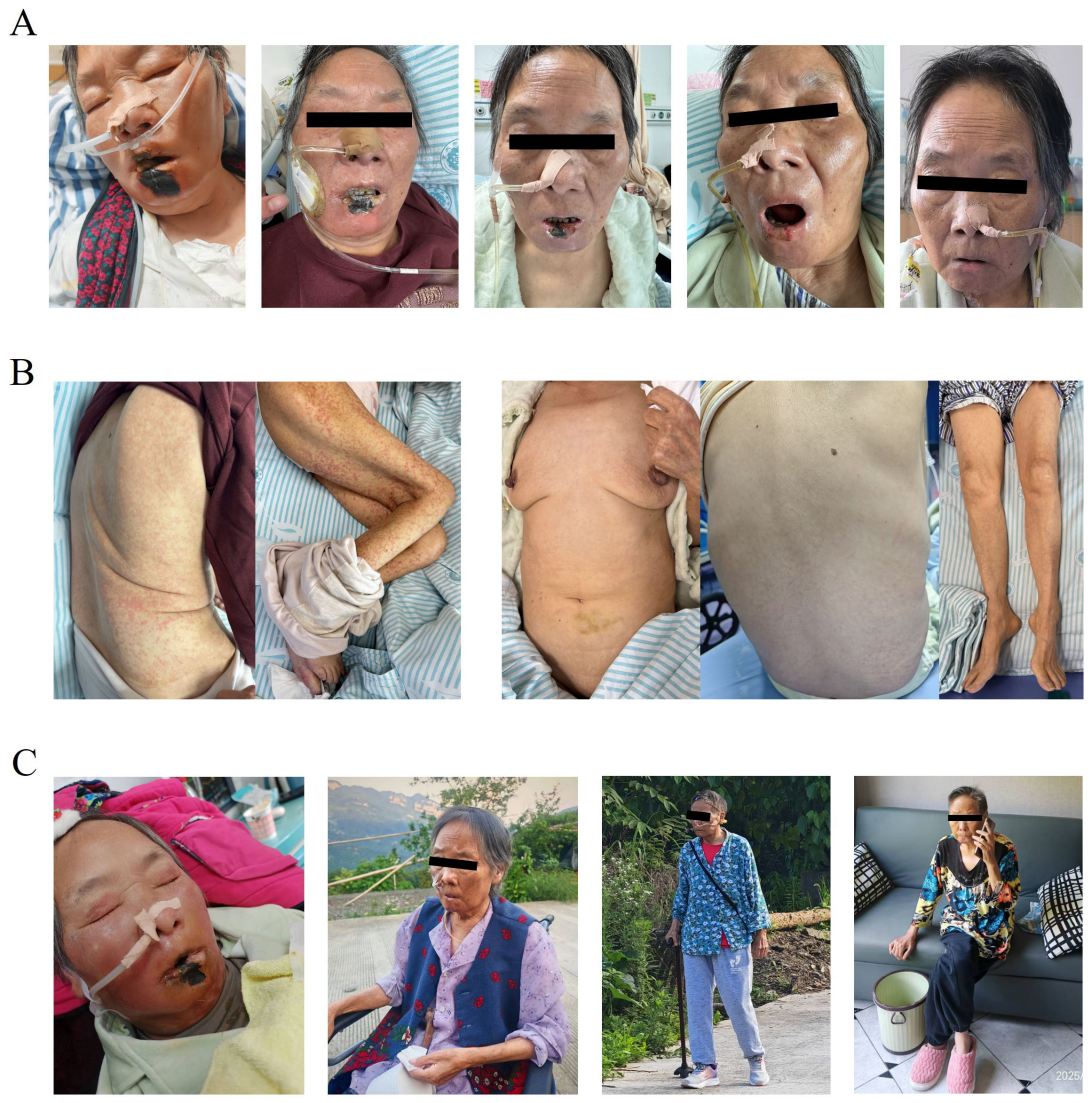
Progressive clinical improvement following treatment. **(A)** Initial presentation showing marked facial and labial edema with prominent necrotic eschar formation on the lips, approximately the size of a quail egg. With ongoing therapy, labial swelling subsided, and the necrotic crusts detached completely. **(B)** Dense erythematous papules and plaques were distributed across the trunk and extremities at onset. Following treatment, lesions gradually faded in color and resolved, with notable alleviation of pruritus. **(C)** The patient was initially bedridden and somnolent. With ongoing treatment, she gradually regained physical function—progressing from assisted bedside ambulation to wheelchair mobility, and eventually walking independently with a cane at follow-up. In parallel, she was able to discontinue nasogastric feeding and resume oral intake autonomously.

A 72-year-old woman presented with a 2-month history of facial erythema and fever, followed by a generalized pruritic papular eruption, without wheals or transient lesions suggestive of urticaria, lasting for over one month. At admission, the patient presented with facial edema, necrotic lip crusting, dysphagia, and a widespread papular rash. DRESS-associated esophageal involvement was clinically suspected as a possible cause of dysphagia; however, endoscopic confirmation was not feasible due to the patient’s critical condition. She had experienced an unintentional weight loss of approximately 20 kg and a persistent high-grade fever up to 39.3 °C. Physical examination revealed cervical lymphadenopathy, lip necrosis, and sacral pressure ulcers. Laboratory investigations demonstrated marked eosinophilia (1.64 × 10^9^/L) and hepatic involvement, including hypoalbuminemia (22.1 g/L). Prior to admission, the patient had a history of polypharmacy with intermittent use of multiple medications prescribed at outside institutions; however, owing to incomplete medication records and the prolonged latency period, a single causative drug could not be definitively identified. Based on the presence of fever, extensive cutaneous eruption, eosinophilia, lymphadenopathy, and internal organ involvement, the patient fulfilled the RegiSCAR criteria for DRESS (**Table 1**). The patient was initially treated with intravenous dexamethasone at a dosage of 10 mg, which was tapered to 7.5 mg on day 2 and further reduced to 5 mg on day 4. In parallel, intravenous immunoglobulin (IVIG) at a dose of 20 g was administered over a 5-day period (**Figure 1**). On day 6, the treatment regimen was modified to include human albumin to correct severe hypoalbuminemia in combination with dexamethasone. Topical therapy consisted of halobetasol propionate cream applied to the trunk and extremities and vitamin E cream for moisturizing. For the lips, epidermal growth factor gel, neomycin sulfate gel, and zinc oxide ointment were used. Due to difficulties in swallowing, the patient was provided with nutritional support through a nasogastric tube, with enteral nutrition administered via a feeding pump. During the treatment course, there was significant improvement in the swelling of the lips, with erythema and papules on the trunk and extremities lightening and darkening. Additionally, pruritus was markedly relieved (**Figure 2B**). On hospital day 9, she developed persistent fever, progressive cytopenias, hyperferritinemia, elevated sCD25, and splenomegaly, meeting six out of eight HLH-2004 criteria (**Table 2**), consistent with a definitive diagnosis of HLH. Given the marked clinical response to corticosteroid therapy, contrast-enhanced CT imaging was performed to evaluate for underlying malignancy, occult infection, and organ involvement, with no evidence of hematologic malignancy or focal infectious source identified. PET-CT was not performed due to clinical instability during the peak of systemic inflammation. Given the rapid progression of hyperinflammation, corticosteroid escalation was prioritized, resulting in prompt defervescence and gradual hematologic recovery. The patient was treated with intravenous dexamethasone at a dosage of 10 mg for its anti-inflammatory effects. During this treatment, the patient’s temperature decreased and remained within the normal range. Hemoglobin and platelet count gradually improved, while ferritin levels progressively decreased (**Supplementary Table S1** for timeline and details). On the 19th day of hospitalization, the patient reported worsening dysphagia, along with new symptoms of paroxysmal headaches and somnolence. Physical examination revealed a suspicious positive Babinski sign on the left side. A lumbar puncture was performed, revealing an intracranial pressure (ICP) of 200 mmH_2_O in the lateral decubitus position. Cerebrospinal fluid (CSF) analysis showed elevated protein levels (0.9 g/L), and ink stain testing was positive. Based on these findings, the treatment regimen was adjusted. Mannitol was administered to reduce ICP. On the first day after the discovery of these symptoms, the patient received amphotericin B (25 mg), fluconazole (100 ml), and dexamethasone (5 mg). From days 2 to 16, the patient continued with amphotericin B 150 mg and fluconazole (100 ml), with dexamethasone (5 mg) continued until day 6, at which point it was discontinued. On day 17, amphotericin B was reduced to 25 mg, while fluconazole (100 ml) continued. During this course of treatment, the patient’s headaches significantly improved, and there was no further somnolence. After one month, the patient showed complete resolution of lip scabs (**Figure 2A**), significant improvement in dysphagia, with the ability to open the mouth sufficiently to eat, and progressive recovery of motor function—from being dependent on a wheelchair to walking with crutches (**Figure 2C**).

**Table 1:** RegiSCAR scoring system in DRESS for the patient.

Criteria	Substract 1 point	0 points	Add 1 point	Add 2 points	Patient score & supporting evidence
Fever (≥ 38.5°C)	No/unknown	Yes			0 (Persistent high-grade fever; Tmax 39.3°C)
Enlarged lymph nodes (≥ 2 sites, ≥ 1 cm)		No/unknown	Yes		1 (Cervical and axillary lymphadenopathy on CT)
Eosinophilia (× 10^9^/L)		No/unknown	≥ 0.7 × 10^9^/L or ≥ 10% if WBC < 4.0 × 10^9^/L	≥ 1.5 × 10^9^/L or ≥ 20% if WBC < 4.0 × 10^9^/L	2 (EOS: 1.64 × 10^9^/L at admission; normal: 0.02–0.52 × 10^9^/L)
Atypical lymphocytosis		No/unknown	Yes		1 (Documented on peripheral blood smear)
Skin rash extent (% body surface area)		No/unknown	> 50%		1 (Rash involved face, trunk, and extremities)
Skin rash suggesting DRESS^a^		Yes/unknown	Yes		1 (Facial edema, infiltrated papules)
Skin biopsy suggesting DRESS	No	Yes/unknown			
Organ involvement Liver Kidney Lung Muscle/heart Pancreas Other organ		No	1 organ	≥ 2 organs	2 (Liver: severe hypoalbuminemia ALB 22.1 g/L, suppressed enzymes; Kidney: acute kidney injury Cr 247 μmol/L)
Rash resolution ≥ 15 days	No/unknown	Yes			0 (Not applicable at time of initial scoring)
Exclusion of other causes^b^		No/unknown	Yes		1 (Negative serologies and cultures for alternative infections)
Total score (maximum = 9)					8 (Definite case)

DIHS, drug-induced hypersensitivity syndrome; DRESS, drug reaction with eosinophilia and systemic symptoms; WBC, white blood cell. Diagnosis made based on total score: < 2 points: not DIHS; 2–3 points: possible DIHS; 4–5 points: probable DIHS; > 5 points: definitive case. Our patient received a score of 8, corresponding to a definite DIHS case. (A) Suggests DRESS if ≥ 2 purpuric lesions, infiltration, facial edema, psoriasiform desquamation. (B) If ≥ 3 negative: antinuclear antibody, blood culture, hepatitis A virus, hepatitis B virus, hepatitis C virus, chlamydia, mycoplasma.

**Table 2:** HLH-2004 diagnostic criteria for the patient.

HLH-2004 diagnostic criteria	Met in patient (Y/N)	Key supporting findings in this case
1. Fever	Yes	Persistent high-grade fever (Tmax 39 °C)
2. Splenomegaly	Yes	Confirmed by imaging (CT scan)
3. Cytopenias (affecting ≥ 2 of 3 lineages): - Hemoglobin < 9.0 g/dl - Platelets < 100 × 10^9^/L - Neutrophils < 1.0 × 10^9^/L	Yes	Progressive decline in hemoglobin and platelets (Hemoglobin: 64 g/L, 73 g/L, 71 g/L. Platelets: 64 × 10^9^/L, 69 × 10^9^/L.)
4. Hypertriglyceridemia (fasting ≥ 265 mg/dl) and/or Hypofibrinogenemia (≤ 150 mg/dl)	No	
5. Hemophagocytosis in bone marrow, spleen, or lymph nodes	Yes	Identified in bone marrow biopsy
6. Low or absent NK-cell activity	No	
7. Ferritin ≥ 500 ng/ml	Yes	Significantly elevated (Strongly Elevated: 542.9 ng/ml, 635.0 ng/ml, 2552 ng/ml, 2772ng/ml).
8. Elevated soluble CD25 (sIL-2 receptor) ≥ 2,400 U/ml	Yes	Extremely Elevated: sIL-2R > 7720.0 IU/ml on multiple tests.

Diagnosis of hemophagocytic lymphohistiocytosis is made when at least 5 of the 8 criteria are fulfilled.

## Discussion

Drug Reaction with Eosinophilia and Systemic Symptoms (DRESS) is a severe hypersensitivity disorder that can progress to HLH through T-cell hyperactivation and subsequent cytokine storm (6). Recent studies (7, 8) have highlighted the complex immunopathogenesis of DRESS, in which dysregulated interactions between innate and adaptive immune responses lead to widespread inflammation and multi-organ involvement. Central to this process is the activation of CD8^+^ T cells, which, upon recognition of drug-modified or stress-induced self-antigens, trigger a cascade of pro-inflammatory cytokines, including IL-6, TNF-α, and IFN-γ (9, 10). Importantly, this immune activation may persist even when the inciting drug cannot be unequivocally identified. In clinical practice, identification of the culprit drug in DRESS is often challenging, particularly in elderly patients with polypharmacy and incomplete medication histories. Delayed-onset presentations further complicate causality assessment, as observed in the present case. As immune dysregulation progresses, HLH—a life-threatening hyperinflammatory syndrome characterized by excessive activation of macrophages and cytotoxic T cells—may develop (11, 12). HLH is associated with markedly elevated ferritin levels and increased soluble immune activation markers, such as soluble IL-2 receptor (sCD25) and soluble CD163 (13, 14). Notably, DRESS and HLH share overlapping immunopathogenic pathways, including sustained T-cell activation, macrophage-driven inflammation, and activation of the NLRP3 inflammasome, ultimately predisposing patients to uncontrolled systemic inflammation and multi-organ failure if not promptly recognized and treated (15, 16).

The evaluation of a febrile patient with hyperferritinemia and cytopenia requires a broad differential diagnosis, extending beyond DRESS-associated HLH. Key considerations include infection-triggered secondary HLH, such as those caused by viral pathogens (EBV, CMV, and HIV), bacterial sepsis, or fungal infections. Notably, tick-borne illnesses, including human granulocytic anaplasmosis (Anaplasma phagocytophilum) and ehrlichiosis, can mimic HLH, highlighting the importance of molecular testing and peripheral smear examination in endemic areas. Non-infectious mimics—such as hematologic malignancies (e.g., lymphoma), autoimmune diseases (e.g., adult-onset Still’s disease, lupus), and other severe drug reactions—must also be excluded (5, 17, 18). A thorough evaluation, including detailed exposure history, serologic and molecular studies, imaging, and often bone marrow examination, is crucial for an accurate diagnosis (5, 19). In the present case, no alternative infectious, malignant, or autoimmune triggers were identified, and the overall clinical presentation was most consistent with DRESS-associated HLH.

In this case, the diagnosis of DRESS complicated by HLH was confirmed by clinical features and laboratory findings, including elevated ferritin levels and abnormal bone marrow findings consistent with hemophagocytosis. This underscores the critical need for early detection and monitoring of systemic inflammation and hematologic indices, particularly ferritin and sCD25, during the course of DRESS. Delayed recognition of HLH can result in a significant worsening of prognosis due to the risk of multi-organ dysfunction and death. Therefore, timely intervention with immunosuppressive therapy, including corticosteroids, is essential to control the cytokine storm and prevent further complications (6). In our patient, escalation of corticosteroid therapy resulted in rapid defervescence and hematologic improvement, supporting an inflammation-driven HLH phenotype.

Additionally, the detection of cryptococcal meningitis in this patient highlights the importance of considering opportunistic infections, particularly in patients receiving immunosuppressive therapy. Cryptococcal meningitis is often a clinically silent infection, especially in immunocompromised individuals, and may present with nonspecific neurological symptoms such as headaches and altered mental status (20). This patient’s diagnosis of cryptococcal meningitis emphasizes the need for early CSF examination and fungal cultures in immunosuppressed patients presenting with neurological symptoms. The use of antifungal therapy, in this case, was pivotal in controlling the infection, and the patient showed significant improvement following treatment (20).

The management of DRESS complicated by HLH and opportunistic infections requires a dynamic, multidisciplinary approach. In this case, immunomodulation through corticosteroids, combined with antifungal therapy and supportive care, was effective in stabilizing the patient. Immunosuppressive therapy, while necessary to control the inflammatory response, must be carefully balanced to avoid exacerbating infection risk (6). Although IL-1 or IL-6 blockade has been reported in refractory or cytokine-driven HLH, the rapid and sustained response to corticosteroids in this patient rendered additional biologic therapy unnecessary at that stage. Moreover, the subsequent development of opportunistic fungal infection further supported a cautious approach to escalation of immunosuppression.

Regarding long-term follow-up and prognosis, it is essential to recognize that patients with DRESS complicated by HLH and opportunistic infections are at risk for prolonged immune dysregulation and potential recurrence of HLH (21–23). Continuous monitoring of hematologic indices, inflammatory markers, and immune function is critical. Long-term follow-up should also include periodic screening for latent infections, such as cryptococcosis and other opportunistic pathogens, given the immunosuppressive nature of the therapy used. Notably, the prolonged inflammatory prodrome and marked steroid responsiveness in this case raise the possibility of an underlying autoinflammatory component; however, inflammasome-focused molecular studies were not performed due to limitations of testing availability in the acute clinical setting, which represents a limitation of this report. In this patient, the current follow-up has been favorable, with significant clinical improvement in neurological and systemic symptoms. The patient’s motor function has improved, and dysphagia has significantly resolved, with the ability to perform oral intake and ambulate with assistance, demonstrating the potential for functional recovery even after severe inflammatory insults. However, the prognosis for such patients remains variable and depends on timely diagnosis, appropriate management, and the ability to mitigate the long-term effects of immunosuppressive therapy. The potential for relapse of HLH or the development of other complications, such as secondary infections or organ dysfunction, necessitates close, long-term surveillance to ensure optimal outcomes.

## Conclusion

This case underscores the complex and often overlapping pathophysiology of DRESS and HLH, highlighting the importance of early recognition, aggressive immunosuppressive therapy, and vigilant monitoring for opportunistic infections. The favorable current outcome of the patient illustrates the potential for recovery with timely intervention, but it also emphasizes the necessity for prolonged follow-up to assess long-term prognosis and address the challenges posed by immunosuppressive therapy and potential relapses.

## Data Availability

The raw data supporting the conclusions of this article will be made available by the authors, without undue reservation.
