# Supplementary material for: Severe DRESS syndrome with hemophagocytic lymphohistiocytosis and cryptococcal meningitis: a case report and diagnostic challenge
Source: Front Immunol. 2026 Jan 30;17:1703040. doi: 10.3389/fimmu.2026.1703040 (PMC12901459; doi:10.3389/fimmu.2026.1703040)
Supplement: Supplementary file 1 [file Table1.docx]

****Supplement Table 1: Timeline of Disease Evolution and Management****

| Timepoint  (Hospital Day) | Key Event / Diagnostic Turnpoint | Primary Clinical Manifestations | Core Laboratory / Imaging Evidence | Treatment Measures & Adjustments |
| --- | --- | --- | --- | --- |
| **Admission (Day 0)** | **DRESS Admission (Initial Treatment)** | Facial and generalized rash, pruritus, submandibular and cervical edema, dysphagia, inability to eat. | Hb 73 g/L; ESO% 22.3%; ESO# 1.64×10⁹/L; Cr 247 μmol/L; Alb 22.1 g/L; IgE >2000 IU/mL. | **Initial Therapy:** Dexamethasone 10mg iv; IVIG 20g iv; Human Albumin; Nasogastric tube nutrition; Topical medications (Halobetasol/Vitamin E/Growth Factor Gel). |
| **Day 6** | **Steroid Taper & Supportive Care** | Condition stable. | Progressive decline in hemoglobin and platelets (Hgb 73-65 g/L, PLT 160-64 x 10⁹/L). | Dexamethasone reduced to 5mg in combination with Human Albumin 10g qd. |
| **~Day 9** | **HLH Diagnosis & Treatment** | Onset of persistent fever. | **Met HLH-2004 Criteria:** 1. Fever; 2. Splenomegaly (imaging); 3. Cytopenias (Hgb 67-73 g/L, PLT 64-69 x 10⁹/L); 4. Ferritin >500 ng/mL (542.9 ng/mL); 5. sCD25 >2400 U/mL (7720 U/mL). | Dexamethasone was increased to 10 mg. In response, the patient's body temperature normalized, blood counts improved, and ferritin levels exhibited a sustained decline. |
| **Day 9-18** | **HLH Treatment Response** | Body temperature normalized. Hb/Plt gradually increased. Ferritin decreased. | **Indicators of Treatment Efficacy:** Normalization of temperature; Trendwise increase in hemoglobin and platelet counts; Progressive decrease in serum ferritin. | Dexamethasone 10mg maintained. |
| **~Day 19** | **Cryptococcal Meningitis Diagnosis** | **New/Worsening:** Headache, somnolence, worsened dysphagia. **Sign:** Suspicious positive left Babinski sign. | **Lumbar Puncture:** Intracranial pressure 200 mmH₂O; CSF protein 0.9 g/L; **Positive India ink stain**. | **Add:** 1. **Mannitol** for reduced ICP. 2. **Antifungals:** Amphotericin B (25mg), Fluconazole (100mL), Dexamethasone 5mg. |
| **Day 20-35** | **Intensified Antifungal Therapy & Recovery** | Headache and somnolence resolved. Complete resolution of lip eschar, able to open mouth and eat. Progressed from bedridden to walking with crutches. | Clinical signs and symptoms continuously improved. | Amphotericin B **increased to 150mg/d**, Fluconazole (100mL). Dexamethasone **discontinued** after 6 days of antifungal therapy. |
| **Day 36+** | **Recovery & Maintenance** | Significant recovery of self-care ability. |  | Amphotericin B **tapered to 25mg/d** for maintenance. |

Footnote to the text regarding Albumin: Human Albumin (10g per dose) was administered on multiple occasions to correct severe hypoalbuminemia, as evidenced by serum albumin levels as low as 22.1 g/L on admission (reference range: 35-52 g/L). This was aimed at maintaining colloidal osmotic pressure and providing nutritional support.
